# Usefulness of real time PCR for the differentiation and quantification of 652 and JP2 Actinobacillus actinomycetemcomitans genotypes in dental plaque and saliva

**DOI:** 10.1186/1471-2334-6-98

**Published:** 2006-06-13

**Authors:** Germano Orrù, Mario Francesco Marini, Maria Laura Ciusa, Daniela Isola, Marina Cotti, Marco Baldoni, Vincenzo Piras, Elisabetta Pisano, Caterina Montaldo

**Affiliations:** 1 O.B.L. (Oral Biotechnology Laboratory), Dipartimento di Chirurgia e Odontostomatologia Università degli Studi di Cagliari, Cagliari, Italy; 2 Universita' degli Studi Milano Bicocca, Dipartimento di Neuroscienze, Dottorato di Ricerca in Parodontologia Sperimentale, Milano, Italy

## Abstract

**Background:**

The aim of our study is to describe a fast molecular method, able to distinguish and quantize the two different genotypes (652 and JP2) of an important periodontal pathogen: *Actinobacillus actinomycetemcomitans*. The two genotypes show differences in the expression of an important pathogenic factor: the leukotoxin (ltx). In order to evidence this, we performed a real time PCR procedure on the ltx operon, able to recognize *Aa *clinical isolates with different leukotoxic potentials.

**Methods:**

The specificity of the method was confirmed in subgingival plaque and saliva specimens collected from eighty-one Italian (Sardinian) subjects with a mean age of 43.9, fifty five (68 %) of whom had various clinical forms of periodontal disease.

**Results:**

This procedure showed a good sensitivity and a high linear dynamic range of quantization (10^7^-10^2 ^cells/ml) for all genotypes and a good correlation factor (R2 = 0.97–0.98). Compared with traditional cultural methods, this real time PCR procedure is more sensitive; in fact in two subgingival plaque and two positive saliva specimens *Aa *was only detected with the molecular method.

**Conclusion:**

A low number of Sardinian patients was found positive for *Aa *infections in the oral cavity, (just 10 positive periodontal cases out of 81 and two of these were also saliva positive). The highly leukotoxic JP2 strain was the most representative (60 % of the positive specimens); the samples from periodontal pockets and from saliva showed some *ltx *genotype for the same patient. Our experience suggests that this approach is suitable for a rapid and complete laboratory diagnosis for *Aa *infection.

## Background

*Actinobacillus actinomycetemcomitans *(*Aa*) is a gram-negative, facultative anaerobe, implicated in numerous human diseases such as periodontitis, endocarditis, meningitis and osteomyelitis. [1-4]. The primary ecological niches of this bacteria, which causes localized aggressive periodontitis, are dental plaque and periodontal pockets and its presence mainly occurs in chronic adult periodontitis [5-7].*Aa *has been studied because it produces a powerful leukotoxin (ltx) able to kill human leukocytes [8-13]. Most of the *Aa *strains isolated from non-diseased periodontal sites produce low levels of leukotoxin, while aggressive forms of *Aa *-associated periodontitis show highly leukotoxic microrganism clones [8,46]. The leukotoxin is expressed by an operon consisting of four genes (*ltx C, ltx A, ltx B, ltx D*); ltx A is the functional toxin while the three remaining genes are required for activating and transporting the leukotoxin [12,14]. Different transcription levels of ltx can depend on a specific 530-bp sequence in the ltx promoter region, which leads to both lower expression and toxicity (strain 652). When this region is deleted (this occurs in strain JP2) there is a faster ltx production which leads to a better protein transcription than in strain 652: this seems to be responsible for the periodontal destruction [15-18].

Standard culture methods used for *Aa *detection in clinical samples have some disadvantages such as the need for a required nutritionally complex media for growth and several days of incubation [19,20]. Molecular assays for *Aa *detection are based on DNA-probes, PCR and real time PCR methods; they are faster and more sensitive than cultural systems, although they do not allow a simultaneous detection and quantization of the different genotypes [21-34]. To overcome these outbreaks we used a real time PCR method for the differentiation and quantization of *Aa *652/JP2 strains, based on the different length of PCR products in the two genotypes; this method is based on the principle that, by using SYBR Green I, the melting temperature (T_m_) of the PCR product gives information about the sequence length and also allows different genotype amplicons to be identified and quantified.

## Methods

### Clinical specimens

Subgingival plaque and saliva samples were collected from 81 male and female subjects, aged from 7 to 81, recruited from the Department of Dental Disease Prevention (University of Cagliari), who had given informed consent to take part in the microbiological analysis. The patient's conditions were: non-diseased (n = 26.32%), with gingivitis (n = 31, 38.3%), with chronic periodontitis (n = 17.21%) and with aggressive periodontitis (n = 7, 8.7%) [35]. Each patient's health status and background were recorded: age, sex, probing pocket depth, clinical attachment level, bleeding on probing (measured in six sites on all teeth present in the mouth). None of the patients who took part in the experiments was under antibiotic therapy during the previous 6 months. At first, 400 μl of saliva was collected from each patient and placed into a sterile tube, afterwards the subgingival plaque samples were obtained (one site per patient) from the deepest periodontal sites in all subjects. The sample area was isolated using sterile cotton rolls and air-dried to avoid saliva contamination; supragingival plaque was removed by using a sterile curette. A sterile paper point ISO 45 (Roeko Dental, Langenau, Germany) was inserted into the pocket and held in place for 30 seconds [36]. The paper point was then removed and placed into a vial containing 800 μl of sterile saline solution, NaCl 0.9%, with 15 glass beads (about 100 mg Bio-Spec Products, Bartesville, USA). After vigorous vortexing, 200 μl of the suspension was immediately used for culture analysis, while the remaining suspension (600 ul) was stored at -20°C and 400 ul of this were used for DNA extraction.

### Positive control strains

#### a) Aa strains

The *Aa *strains used were: (i) CCUG 37005 (Culture Collection, University of Göteborg, Sweden, genotype 652) and (ii) clinical isolate, strain GO1 (genotype JP2). The two strains were maintained at -80°C in vials containing Schaedler Broth with 15% glycerol and cultured in Columbia agar blood (Microbiol, UTA, Cagliari, Italy) at 37°C with 5% CO_2 _in jar (Biomérieux Marcy l'Etoile, France). After 1 week of incubation, colonies of each genotype were suspended in a sterile saline solution (NaCl 0.9%) to obtain a concentration of 1 McFarland scale 3*10^8 ^cells/ml (counted with the McFarland method) these suspensions were used as follows:

1. to assess specificity: we prepared different suspensions in 400 μl of sterile saliva (obtained by filtration with a 0.5 μm filter, Millipore Molseim, France), (i) containing 10^6 ^CELLS/ml of each genotype and (ii) tubes with different proportional mixtures of the two genotypes, 1/2, 1/4, 1/8, 1/10 652/JP2 or *vice versa*)

2. to assess sensitivity: we prepared 10 fold serial dilutions of each genotype in sterile saliva, ranging from 10^7 ^-10^1 ^cells/ml.

The exact bacterial concentration of these standards was obtained by colony forming unit (CFU) plate counted in Columbia agar blood (Microbiol, Uta, Cagliari, Italy. These suspensions served as a standard for measuring the method sensitivity and for the quantification curve after DNA extraction.

#### b) Other periodontal bacteria used as positive control

To assess the role of other periodontal pathogens in these samples, a traditional PCR was used with subsequent positive controls: *Porphyromonas gingivalis *CCUG 25893, *Prevotella intermedia *CCUG 24041, (Culture Collection, University of Göteborg, Sweden), *Tannerella forsythensis *cip 105220 (Institut Pasteur, Paris, France) *Treponema denticola *DSMZ 14222-Deutsche *Sammlung von Mikroorganismen*, Braunschweig, Germany. These strains (except *T.denticola*) were cultured on Shaedler Anaerobe Agar plates (Microbiol, Uta, Cagliari, Italy), incubated in an anaerobic jar for 7 days at 37°C. 1 ml of these bacterial suspensions was used for DNA extraction.

### DNA extraction

Genomic DNA from positive controls and clinical samples was obtained by the CTAB modified method. 400 μl of sample were added to 70 μl of 10% sodium dodecyl sulphate (SDS) and 5 μl of proteinase K at 10 mg/ml concentration (SIGMA – Aldrich, ST. Louis, Missouri, USA); after vigorous vortexing, this mixture was incubated for 10 minutes at 65°C. Next, 100 μl of NaCl [5 M] and 100 μl of CTAB/NaCl (0.274 M CTAB, Hexadecyl trimetylammonium bromide and 0.877 M NaCl, Sigma-Aldrich) were added to the tube, which was vortexed briefly and incubated at 65°C for 10 minutes. 750 μl of SEVAG (Chloroform: Isoamyl Alcohol 24:1, Sigma-Aldrich) were added and the mixture was vortexed for 10 sec. After centrifuging for 5 min (at 12000 rpm) 0.6 volumes of isopropanol (Sigma-Aldrich) were added to the supernatant. After 30 min at -20°C and after being centrifuged for 30 min at 12.000 rpm, the pellet was dried at room temperature for 20 min and suspended in 20 μl of molecular biology grade distilled water (Gibco, Invitrogen Paisley, Scotland, UK). 2 μl of this were used as DNA suspension for conventional PCR and real time PCR reaction.

### Primers design

**Figure 1:**
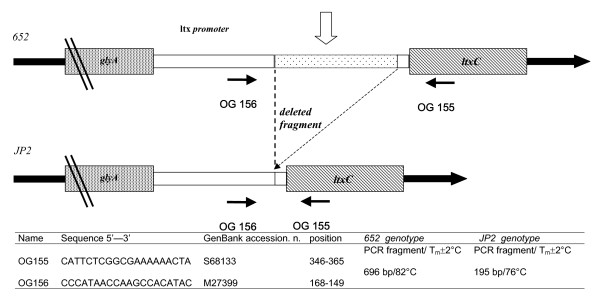
**Structure of Real time PCR design for *Actinobacillus actinomycetemcomitans *652, JP2 genotype detection**. Primers were designed to circumscribe a fragment of 530 bp present in 652 but deleted in the JP2 strain. The two different PCR products showed 6 °C difference in T_m_s after PCR real time melting curve analysis.

Primers for real time PCR were designed using the promoter region on the Leukotoxin operon sequences extracted from the NCBI database GenBank with S68133 and M27399 accession numbers. Figure 1 shows primer sequence and position and the real time PCR mechanism with the two different (652/JP2) Aa genotypes. Possible oligonucleotide dimer formation, self-complementarity and the annealing temperatures of the real time PCR were calculated using the Oligo program vers. 4 (MedProbe, Oslo, Norway). The real time PCR primers (OG155 and OG156) amplified a region of 696 bp in the 652 genotype and 195 bp in the JP2 genotype (Figure 1). The theoretic melting temperatures of the different PCR amplicons (Tms) were calculated using module 1 of the DNA hybridization prediction algorithm program "HYTHER" [37,50] with the following sets of parameters: (i) monovalent cation concentration at 0.05 mol/L, (ii) Mg^2+ ^at 0.004 mol/L, (iii) a concentration of PCR products (Top/Bottom strands) at 10^-7 ^mol/L and (iv) hybridisation temperature at 37°C. The same procedures have been used to design all the oligonucleotides used in traditional PCR (Table 1).

**Table 1:** Primers used and strains detected by conventional PCR.

**Name**	**Target**	**Strain**	**Sequence 5'--------------3'**	**GenBank Accession No**	**PCR product (bp)**
OG 43	*16S rRNA*	*Actinobacillus actimycetemcomitans*	CCAAGTGTGATTAGGTAGTT	M75036	226
OG 44	*16S rRNA*	*Actinobacillus actimycetemcomitans*	ACCAACCTTCCTCAATAC	M75036	
OG53	*16S rRNA*	*Prevotella intermedia*	CGTATCCAACCTTCCCTCC	X73965	389
OG54	*16S rRNA*	*Prevotella intermedia*	ATTAGCCGGTCCTTATTCGAAG	X73965	
OG94	*prtC*	*Porphyromonas gingivalis*	GAATCAAATACTTCAGCCGTCT	AB006973	143
OG95	*prtC*	*Porpyromonas gingivalis*	TTGCAGTTCGTATCGGATCT	AB006973	
OG45	*16S rRNA*	*Tannerella forsythensis*	GTCGGACTAATACCTCATAAAACA	L16495	222
OG46	*16S rRNA*	*Tannerella forsythensis*	TCGCCCATTGACCAATATT	L16495	
OG348	*16S rRNA*	*Treponema denticola*	AGAGAAAGGGTAATTTGAAG	AF179264	399
OG349	*16S rRNA*	*Treponema denticola*	TATTATTGTCCCTTCTTTCTT	AF179264	

### Real time PCR

Real time PCR was performed with a LightCycler instrument and a LightCycler DNA Master SYBR Green I kit (Roche Diagnostics Mannheim, Germany), according to the manufacturer's instructions. The 20 μl final volume contained 4 mM MgCl_2_, 1 μM of each primer (OG155-OG156) and 2 μl of DNA extract. The PCR program was: (i) denaturation at 95°C for 30 sec, (ii) 40 cycles of: 1 sec at 95°C, 10 sec at 49°C, 40 sec at 72°C and 3 sec at 74.3°C. (iii) The melting curve was performed for 0 seconds at 95°C, 45°C, 95°C. Transition rates were: 5°C/s in 72°C segment, 0.1°C/s in 45 °C segment and 20 °C/s for other steps. Fluorescence was detected at the end of the 74.3°C segment in the PCR step (single mode), and at 45°C segment in the melting step (continuous mode) in the F1 channel. During the initial optimization of the real time reaction, products were analyzed using agarose gel to ensure a correct sample product size. After real time PCR, samples were recovered from capillaries by reverse centrifugation into microcentrifuge tubes (Eppendorf 0.2 μl), and mixed with blue loading buffer and finally 10 μl of each sample were utilized in 1% agarose electrophoresis gel (Invitrogen, Palsley, Scotland, UK) and stained with ethidium bromide.

### Differentiation between JP2 and 652 genotypes

By using two primers (OG155 and OG156) flanking an ltx promoter region, we obtained two different length PCR products: 696 bp for 652 and 195 bp for the JP2 genotype. These amplicons showed two different T_m_s melting peaks after the PCR real time reaction with SYBR green I dye. To evaluate product specificity, ltx PCR amplicons were also sequenced by a conventional automated sequencer as described in literature by Ianelli et al.,1998, [38]. The results were edited and analyzed with nucleotide-nucleotide BLAST (blastn) [51] and compared with sequences deposited in the DNA data bank.

### Expression of the Aa concentration in dental plaque and saliva

Real time PCR standard curves were performed on different DNA extracts, obtained by different *Aa *genotype suspensions with a concentration range of 10^7^-10^1 ^cells/ml. The number of bacteria was calculated by the interpolation of the clinical sample threshold cycle [39] with a standard curve obtained for each genotype (Figure 4). Bacterial concentration in specimens was expressed in: i) *Aa *cells/ml paper point suspension for subgingival plaque and ii) *Aa *cells/ml saliva in saliva samples. We used the following equation to calculate *Aa *concentration for the paper point specimens.

a) [cells/ml paper point suspension].= ([*Aa *Rt cells]*2,5.

[Aa Rt cells]= Bacterial cells, calculated by PCR real time standard curve interpolation.

### Cultural method

For a sensitivity study, molecular methods were compared with traditional culture analysis for all the clinical samples: 200 μl of saliva and paper point suspensions were diluted 10 and 100 fold in Schaedler Broth (Microbiol, Uta, Cagliari, Italy). 100 μl of each dilution were plated in Columbia agar blood (Microbiol, Uta, Cagliari, Italy) and incubated in a 15% CO_2 _atmosphere at 37°C in a jar using the Genbox system (Biomérieux Marcy l'Etoile, France). After 6 days of incubation the typical *Aa *colonies were identified by a biochemical test using API 20A (Biomérieux Marcy l'Etoile, France) according to the manufacturer's instructions.

### Conventional PCR

For the 81 analyzed samples we used traditional PCR able to detect: (i) *A. actinomycetemcomitans *by different DNA target (16S rRNA) and (ii) *P. gingivalis, P. intermedia*, *T. forsythensis*, *T. denticola*. For all bacteria, the reaction was performed in 25 μl reaction volumes using MegaMix 2MM-5 (Microzone Limited, West Sussex, UK) according to the manufacturer's instructions. The primer and target sequences used are shown in Table 1. The mixture contained 7 pmol of each primer, 3.8 mM MgCl_2 _and 2 μl of DNA suspension. The thermocycler profile was as follows: an initial denaturation at 95° C for 5 min; 35 cycles consisting of 50°C for 1 min, 68° C for 3 min and 40 sec and 95° C for 1 min. PCR products were analysed by electrophoresis on a 1.2 % agarose gel containing ethidium bromide (0.5 mg/ml). With reconstruction experiments this method showed a detection limit range of 10-50 cells/PCR.

## Results

### Reconstruction experiments: specificity and sensitivity of the method

**Figure 2:**
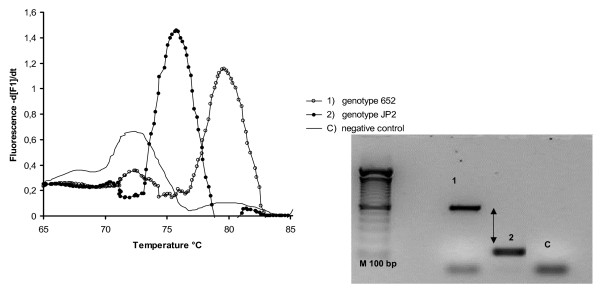
**Real time PCR melting curve and agarose gel electrophoresis results obtained from saliva reconstruction samples containing different *Actinobacillus actinomycetemcomitans *genotypes**. Curves 1–2: using a suspension containing the 652 or JP2 *Aa *genotype, the difference in T_m_s melting peaks was 6°C between the two clones. The presence of a single 696 bp band for 652 and 192 bp for the JP2 strain in 1% agarose electrophoresis gel (Metaphor, BMA, Rockland ME USA) suggests a total absence of non-specific products, gel bands 1–2. Melting peak at 73°C in negative control and the small band in the control samples "C" are primer dimer products.

**Figure 3:**
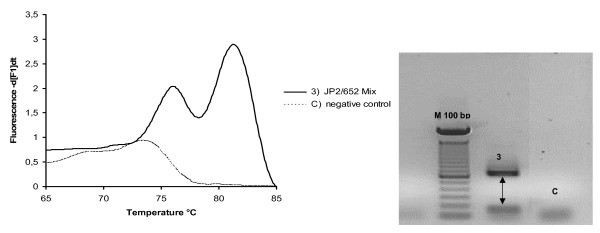
**Real time PCR melting curve and agarose gel electrophoresis results obtained using a suspension containing a mixture of two genotypes: **a single curve is obtained with two distinct T_m_s' peaks which confirm good system selectivity. Melting peak at 73°C in negative control and the small band in the control samples "C" are primer dimer products.

**Figure 4:**
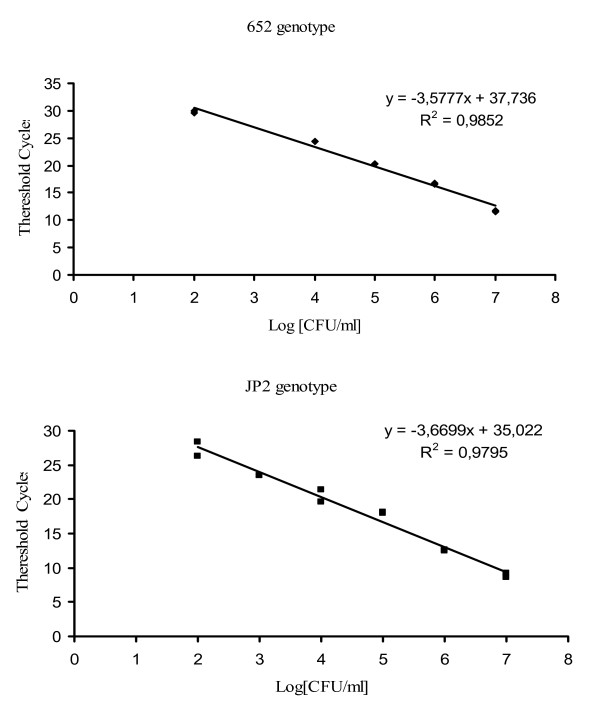
**Quantitative standard curves**. Linear regressions obtained by real time PCR using a LightCycler instrument with SYBR Green I protocol of serially diluted suspensions for JP2 or 652 *Actinobacillus actinomycetemcomitans *genotypes (10^7^-10^2 ^cells/ml). Threshold cycle (CT) value is directly related to the amount of PCR product and therefore related to the initial amount of target DNA present in the PCR reaction. CT (threshold cycle) represent the fractional PCR cycle number at which the fluorescence signal exceeds background fluorescence [40].

A reconstruction experiment confirmed the PCR real time selectivity in the DNA extracts of three different suspensions: (i) *Aa *652, (ii) *Aa *JP2 and (iii) a mixture of the two genotypes. Figures 2, 3 shows melting curves and the 1% agarose gel electrophoresis results of PCR real time products. In our study, with reconstruction and with clinical samples, we demonstrated melting temperatures of 76 ± 2 °C in the JP2 positive samples, and 82 ± 2 °C in the 652 positive samples; a double melting peak was obtained with both genotype mixtures tested (Figure 3). The 6 °C difference in T_m_s allows an easier differentiation of two genotype profiles; no cross-reaction in T_m_s peaks was detected with strains 652 and JP2, indicating the good selectivity of the method. Only one size band for each genotype was verified and confirmed in agarose gel electrophoresis, respectively 696 bp for 652 and 192 bp for the JP2 strain (Figure 2); this suggests a total absence of non-specific products. Real-time PCR, as evaluated here, has been shown to have a detection limit of 40 *Aa *652 or JP2 cells/PCR (100 cells/ml). The wide linear range (10^7^-10^2^cells obtained for two genotypes) as illustrated in Figure .4, indicates the efficiency of this real time PCR. Standard curves showed a similar slope and a good correlation regression coefficient R2 of 0.98–0.97 [40], (Figure 4). In these experiments negative control contained DNA saliva extracts recovered from a non-diseased patient with PCR/negative culture for *Aa*, the total DNA amount of these samples was about 50 μg/μl. C lanes/curves do not contain aspecific products from previous interaction with human or non-specific bacterial genomic DNA as shown in Figures 2, 3. The DNA sequencing results of the PCR amplicons were a perfect match, with Blast program analysis [51], for the 652 genotype (Gen Bank accession n. S68133) or the JP2 genotype (Gen Bank accession n. X16829).

### Results with clinical samples

We analyzed plaque and saliva samples collected from 81 patients following the protocols just described. In comparison with the cultural procedure, real time resulted as being a little more sensitive (1 saliva and 2 paper point samples were positive only with molecular methods), while real time and conventional PCR were in accordance. All negative samples with the real time PCR procedure also resulted negative with cultural and conventional PCR methods too. Only one subject in the non-diseased group (26 in total) resulted 652 *Aa *genotype-positive for the periodontal pocket specimen, with 10^3 ^cells/ml bacteria in the paper point suspension and negative in the saliva sample. Table 2 shows that 9 out of 55 diseased patients (16.3%) were infected with *Aa *and the disease severity was associated with a high bacteria title and/or leukotoxic genotype presence. In accordance with scientific data, we only identified the leukotoxic clone (JP2) in patients with severe forms of periodontitis [41]. However, whenever a similar degree of clinical infection was present, and only the leukotoxic 652 genotype was identified, high bacteria titles were present (about ≥ 10^4 ^cells/ml in the paper point suspension). Two patients, with aggressive periodontitis, showed bacteria in saliva samples too; one was found positive for the 652 strain and the other for JP2, the same genotypes were detected respectively in periodontal pockets.

**Table 2:** Real Time PCR results (title and genotype) in comparison with cultural and conventional PCR methods using saliva and subgingival plaque from 10 Sardinian subjects out of 81, positive for *A. actinomycetemcomitans*.

Patient	Disease type	Subgingival plaque			Saliva	
		*Aa *Real time PCR [cells/ml paper point]	Culture for *Aa*	Conventional PCR (for 4 periodontal pathogens)	Real time PCR *Aa *cells/ml	Aa Culture CFU/ml *vs *Conventional PCR (Aa 16S rRNA only)

C1	aggressive periodontitis	1,9*10^4 ^(652)	posit	*Aa*	3,0*10^2 ^(652)	1,5*10^1^/pos
C2	aggressive periodontitis	4,0*10^3 ^(JP2)	posit	*Aa, Pg*	1,3*10^2 ^(JP2)	neg/pos
C4	gingivitis	3,6*10^3 ^(652)	posit	*Aa*,	Nd	neg/neg
C5	chronic periodontitis	6,4*10^3 ^(JP2)	posit	*Aa,Tf*	Nd	neg/neg
C6	chronic periodontitis	1,9*10^3 ^(JP2)	neg	*Aa,Tf*	Nd	neg/neg
C7	aggressive periodontitis	2,8*10^4 ^(JP2)	posit	*Aa,Pi,Tf*	Nd	neg/neg
C8	aggressive periodontitis	4,4*10^4 ^(JP2)	posit	*Aa*,	Nd	neg/neg
C25	aggressive periodontitis	2,0*10^3 ^(JP2)	posit	*Aa*,	Nd	neg/neg
C75	aggressive periodontitis	3,0 *10^4 ^(652)	posit	*Aa, Pg, Pi,Tf*	Nd	neg/neg
C80	no disease	1,0*10^3 ^(652)	neg	*Aa, Pg, Pi,Tf*	Nd	neg/neg

Legend:

*Aa = A. actinomycetemcomitans, Pg = P. gingivalis, Pi = P. intermedia, Tf = T. forsythensis*.

Nd = non-detectable (<10^2 ^cells/ml)

### Subgingival distribution of different periodontal bacteria

Five periodontopathic bacteria: *A. actinomycemcomitans P. gingivalis, P. intermedia, T. forsytensis *and *T. denticola *were detected by a conventional PCR method from the subgingival plaque of all 81 patients. No periodontopathogenic bacterial DNA was observed in 39 of the samples (51%). The percentage of PCR positive samples was: *Aa *12.2%, *Pg *14.8%, *Pi *22%, *Tf *(the most representative bacteria in these samples) 40.8% and *Td *8.7 %. Six specimens resulting positive for *Aa*, (C2, C5, C6, C7, C75, C80) were associated with at least another periodontal bacteria. The principal pathogen was *T. forsythensis *present in 50% of the *Aa *positive samples. Sample C80, isolated in a non-diseased patient (2 mm subgingival pocket), contained all 4 tested pathogens (Table 2). In the PCR results four samples showed *Aa *DNA only (samples C1, C4, C8, C25) and these results were in accordance with the PCR real time and cultural result s(Table 2).

## Discussion

Periodontitis is an inflammatory disease caused by different species of anaerobic bacteria and is the most prevalent human disease; in addition a growing number of studies indicate that severe chronic forms of this disease are not only localized infections, but may also increase of the risk of various systemic conditions such as cardiovascular disease [4,42].

The multi-factoriality of this infection is due to a complex bacteria population with high dynamicity and adaptability; consequently, treating periodontitis is difficult since the elimination of this pathogenic bacteria, once established in periodontal pockets, may not be possible, even with repetitive treatments. Moreover costs related to maintaining and replacing restoration work like fillings and crowns and those of periodontal treatment are high and similar problems occur with dental implants.

For these reasons in periodontal disease prevention, diagnostic methods able to evaluate the virulence factors of periodontal bacteria are important, particularly in subjects without clinical symptoms but infected with these pathogens; for example patient C80 in our study.

81 patients (diseased and non diseased) showed a sub-gingival distribution of periodontal bacteria in accordance with published data [43]. In fact, at least 1 member of the red complex bacteria (*Pg*, *Tf, Td*) 47.2% or orange complex (Pi) 25.9% was found in the majority of diseased patients; ten subjects (0.12%) contained *A. actinomicetemcomitans *in the periodontal pocket and two of these also in saliva [44].

Different studies have considered *Aa *as an important etiological microorganism involved in aggressive periodontitis [16,35,41,45,46] and it has been shown that a high-leukotoxin-producing strain of *Aa *is mainly found among individuals with severe forms of periodontitis [14-16,41]. Different authors have recently described PCR real time methods for *Aa *quantization [26,27,34,47,48], but with current molecular methods it is not possible to obtain simultaneous bacterial quantization and genotypization.

We have described a fast molecular procedure (2 hours) with good sensitivity and specificity, able to quantify and differentiate strains 652 and JP2 of *Aa *simultaneously. We analyzed subgingival plaque and saliva samples collected from subjects with different periodontal status and the results showed that in comparison with recent bibliographic data obtained in other geographical regions, the same differences in *Aa *distribution were also evaluated in Sardinian people [16,49]. In fact, within the non-diseased patients (n= 26, 3.8%), only one individual resulted *Aa *positive with all the three methods used; 24 chronic and 8 subjects with aggressive periodontitis (33%) showed the bacteria in oral clinical samples. Genotype JP2 was the most represented in all periodontal patients whose age was >29 mean = 47. None of the adolescent subjects examined in this study (n= 5, age 10 to 15 years) showed periodontal disease.

In comparison with the other periodontal pathogens tested, 4 samples were positive only for Aa; this could mean that in these samples disease severity may depend on the number and genotype of this microorganism. However when other periodontal pathogens were present, high numbers or high leukotoxic *Aa *genotype were mostly correlated with severe forms of periodontitis. (chronic or aggressive). The presence of this strain in saliva too, (two cases in these patients), has been described by other authors [44]; Our results shows that the same genotype is present in saliva and in periodontal pockets, indicating a possible and continuous bacteria replacement in saliva from an important reservoir which is the periodontal district. Moreover, real time PCR quantification of saliva could explain its role as a means of bacterial transmission in patients with a high infectious dose of *Aa*.

## Conclusion

Experimental results suggest the importance of obtaining both bacterial titre and genotype identification to give the correct microbiological diagnosis in periodontal infections. Further investigations and sample enlargements could give us more answers about the correlation between bacterial status in the oral cavity and disease severity and progression. The presented method/approach is suitable for a rapid and complete laboratory diagnosis of *Aa *infection.

## Abbreviations

*Aa*, *Actinobacillus actinomycetemcomitans; *T_m_, melting temperature; CTAB, Hexadecyl trimetylammonium bromide

## Competing interests

The author(s) declare/s that they have no competing interests.

## Authors' contributions

**GO**: designed the study, primary author of the manuscript. **MFM, MC**: examined and selected the patients, critical appraisal and editing of the manuscript. **MLC, DI**: maintained the isolates, the strains, prepared cultures and performed the real time PCR experiments. **VP, MB**: critical analysis of the manuscript, editing and final presentation. **EP**: assisted in writing the paper and critical analysis of the manuscript. **CM**: analysed the experimental data and critical analysis of the manuscript. All authors read and approved this manuscript.

## Pre-publication history

The pre-publication history for this paper can be accessed here:



## References

[B1] Meyer DH, Fives-Taylor PM (1997). The role of *Actinobacillus actinomycetemcomitans *in the pathogenesis of periodontal disease. Trends Microbiol.

[B2] Paju S, Carlson P, Jousimies-Somer H, Asikainen S (2000). Heterogeneity of *Actinobacillus actinomycetemcomitans *strains in various human infections and relationships between serotype, genotype, and antimicrobial susceptibility. J Clin Microbiol.

[B3] Paturel L, Casalta JP, Habib G, Nezri M, Raoult D (2004). *Actinobacillus actinomycetemcomitans *endocarditis. Clin Microbiol Infect.

[B4] Rottman M, Hanau-Bercot B, Fiszbin M, Raskine L, Gravisse J, Caulin C, Sanson-Le Pors MJ (2002). Diagnosis of *Actinobacillus actinomycetemcomitans *infective endocarditis after steadily negative blood cultures. J Infect.

[B5] Armitage GC (1999). Development of a classification system for periodontal diseases and conditions. Ann Periodontol.

[B6] Tinoco EM, Sivakumar M, Preus HR (1998). The distribution and transmission of *Actinobacillus actinomycetemcomitans *in families with localized juvenile periodontitis. J Clin Periodontol.

[B7] Zambon JJ (1985). *Actinobacillus actinomycetemcomitans *in human periodontal disease. J Clin Periodontol.

[B8] Kachlany SC, Fine DH, Figurski DH (2000). Secretion of RTX leukotoxin by *Actinobacillus actinomycetemcomitans*. Infect Immun.

[B9] Kachlany SC, Fine DH, Figurski DH (2002). Purification of secreted leukotoxin (LtxA) from *Actinobacillus actinomycetemcomitans*. Protein Expr Purif.

[B10] Lally ET, Golub EE, Kieba IR, Taichman NS, Rosenbloom JJ, Rosenbloom CC, Gibson W, Demuth DR (1989). Analysis of the *Actinobacillus actinomycetemcomitans *leukotoxin gene. Delineation of unique features and comparison to homologous toxins. J Biol Chem.

[B11] Lear JD, Karakelian D, Furblur U, Lally ET, Tanaka JC (2000). Conformational studies of *Actinobacillus actinomycetemcomitans *leukotoxin: partial denaturation enhances toxicity. Biochim Biophys Acta.

[B12] Narayanan SK, Nagaraja TG, Chengappa MM, Stewart GC (2002). Leukotoxins of gram-negative bacteria. Vet Microbiol.

[B13] Yamaguchi N, Kubo C, Masuhiro Y, Lally ET, Koga T, Hanazawa S (2004). Tumor necrosis factor alpha enhances *Actinobacillus actinomycetemcomitans *leukotoxin-induced HL-60 cell apoptosis by stimulating lymphocyte function-associated antigen 1 expression. Infect Immun.

[B14] Brogan JM, Lally ET, Poulsen K, Kilian MD (1994). Regulation of *Actinobacillus actinomycetemcomitans *leukotoxin expression: analysis of the promoter regions of leukotoxic and minimally leukotoxic strains. Infect Immun.

[B15] Haraszthy VI, Hariharan G, Tinoco EM, Cortelli JR, Lally ET, Davis E, Zambon JJ (2000). Evidence for the role of highly leukotoxic *Actinobacillus actinomycetemcomitans *in the pathogenesis of localized juvenile and other forms of early-onset periodontitis. J Periodontol.

[B16] Haubek D, Westergaard J (2004). Detection of a highly toxic clone of *Actinobacillus actinomycetemcomitans *(JP2) in a Moroccan immigrant family with multiple cases of localized aggressive periodontitis. Int J Paediatr Dent.

[B17] Hritz M, Fisher E, Demuth DR (1996). Differential regulation of the leukotoxin operon in highly leukotoxic and minimally leukotoxic strains of *Actinobacillus actinomycetemcomitans*. Infect Immun.

[B18] Kolodrubetz D, Spitznagel J, Wang B, Phillips LH, Jacobs C, Kraig E (1996). cis Elements and trans factors are both important in strain-specific regulation of the leukotoxin gene in *Actinobacillus actinomycetemcomitans*. Infect Immun.

[B19] Holm A, Rabe P, Kalfas S, Edwarsson S (1987). Improved selective culture media for *Actinobacillus actinomycetemcomitans *and *Haemophilus aphrophilus*. J Clin Microbiol.

[B20] Slots J (1982). Selective medium for isolation of *Actinobacillus actinomycetemcomitans*. J Clin Microbiol.

[B21] Doungudomdacha S, Rawlinson A, Douglas CW (2000). Enumeration of *Porphyromonas gingivalis*, *Prevotella intermedia *and *Actinobacillus actinomycetemcomitans *in subgingival plaque samples by a quantitative-competitive PCR method. J Med Microbiol.

[B22] Komiya A, Kato T, Nakagawa T, Saito A, Takahashi J, Yamada S, Okuda KA (2000). Rapid DNA probe method for detection of *Porphyromonas gingivalis and Actinobacillus actinomycetemcomitans*. J Periodontol.

[B23] Kuboniwa M, Amano A, Kimura KR, Sekine S, Kato S, Yamamoto Y, Okahashi N, Iida T, Shizukuishi S (2004). Quantitative detection of parodontal pathogens using real-time polymerase chain reaction with TaqMan probes. Oral Microbiol Immunol.

[B24] Lau L, Sanz M, Herrera D, Morillo JM, Martin C, Silva A (2004). Quantitative real-time polymerase chain reaction versus culture: a comparison between two methods for the detection and quantification of *Actinobacillus actinomycetemcomitans*, *Porphyromonas gingivalis *and *Tannerella forsythensis *in subgingival plaque samples. J Clin Periodontol.

[B25] Leys EJ, Griffen AL, Strong SJ, Fuerst PA (1994). Detection and strain identification of *Actinobacillus actinomycetemcomitans *by nested PCR. J Clin Microbiol.

[B26] Maeda H, Fujimoto C, Haruki Y, Maeda T, Kokeguchi S, Petelin M, Arai H, Tanimoto I, Nishimura F, Takashiba S (2003). Quantitative real-time PCR using TaqMan and SYBR Green for *Actinobacillus actinomycetemcomitans, Porphyromonas gingivalis, Prevotella intermedia*, tetQ gene and total bacteria. FEMS Immunol Med Microbiol.

[B27] Morillo JM, Lau L, Sanz M, Herrera D, Silva A (2003). Quantitative real-time PCR based on single copy gene sequence for detection of *Actinobacillus actinomycetemcomitans *and *Porphyromonas gingivalis*. J Periodontal Res.

[B28] Morillo JM, Lau L, Sanz M, Herrera D, Martin C, Silva A (2004). Quantitative real-time polymerase chain reaction based on single copy gene sequence for detection of parodontal pathogens. J Clin Periodontol.

[B29] Poulsen K, Ennibi OK, Haubek D (2003). Improved PCR for detection of the highly leukotoxic JP2 clone of *Actinobacillus actinomycetemcomitans *in subgingival plaque samples. J Clin Microbiol.

[B30] Rudney JD, Chen R, Pan Y (2003). Endpoint quantitative PCR assays for *Bacteroides forsythus, Porphyromonas gingivalis*, and *Actinobacillus actinomycetemcomitans*. J Periodontal Res.

[B31] Saddi-Ortega L, Carvalho MA, Cisalpino PS, Moreira ES (2002). *Actinobacillus actinomycetemcomitans *genetic heterogeneity: amplification of JP2-like ltx promoter pattern correlated with specific arbitrarily primed polymerase chain reaction (AP-PCR) genotypes from human but not marmoset Brazilian isolates. J Microbiol.

[B32] Sanz M, Lau L, Herrera D, Morillo JM, Silva A (2004). Methods of detection of *Actinobacillus actinomycetemcomitans*, *Porphyromonas gingivalis *and *Tannerella forsythensis *in parodontal microbiology, with special emphasis on advanced molecular techniques: a review. J Clin Periodontol.

[B33] Suzuki N, Nakano Y, Yoshida Y, Ikeda D, Koga T (2001). Identification of *Actinobacillus actinomycetemcomitans *serotypes by multiplex PCR. J Clin Microbiol.

[B34] Yoshida A, Suzuki N, Nakano Y, Oho T, Kawada M, Koga T (2003). Development of a 5' fluorogenic nuclease-based real-time PCR assay for quantitative detection of *Actinobacillus actinomycetemcomitans *and *Porphyromonas gingivalis*. J Clin Microbiol.

[B35] Wiebe CB, Putnins EE (2000). The periodontal disease classification system of the American Academy of Periodontology -an update. J Can Dent Assoc.

[B36] Hartroth B, Seyfahrt I, Conrads G (1999). Sampling of periodontal pathogens by paper points: evaluation of basic parameter. Oral Microbiol Immunol.

[B37] Bommarito S, Peyret N, Santa Lucia J (2000). Thermodynamic parameters for DNA sequences with dangling ends. Nucleic Acids Res.

[B38] Iannelli F, Giunti L, Pozzi G (1998). Direct sequencing of long polymerase chain reaction fragments. Mol Biotechnol.

[B39] Rasmussen R, Meuer S, Wittwer C, Nakagawara K (1998). Quantification on the LightCycler. Rapid Cycle Real-Time PCR Methods and Applications.

[B40] Wittwer C, Meuer S, Wittwer C, Nakagawara K (2001). Rapid Cycle Real Time PCR: Methods and Applications. Rapid Cycle Real-Time PCR Methods and Applications.

[B41] Cortelli SC, Jorge AO, Cortelli JR, Jordan SF, Haraszthy V (2003). Detection of highly and minimally leukotoxic *Actinobacillus actinomycetemcomitans *strains in patients with parodontal disease. Pesqui Odontol Bras.

[B42] Beck JD, Offenbacher S (2005). Systemic effects of periodontitis epidemiology of periodontal disease and cardiovascular disease. J Periodontol.

[B43] Socransky SS, Haffajee AD, Cugini MA, Smith C, Kent RL (1998). Microbial complexes in subgingival plaque. J Clin Periodontol.

[B44] Tamura K, Nakano K, Hayashibara T, Nomura R, Fujita K, Shintani S, Ooshima T (2005). Distribution of 10 periodontal bacteria in saliva samples from Japanese children and their mothers. Arch Oral Biol.

[B45] Cao SL, Progulske-Fox A, Hillman JD, Handfield M (2004). In vivo induced antigenic determinants of *Actinobacillus actinomycetemcomitans*. FEMS Microbiol Lett.

[B46] Yang HW, Asikainen S, Dogan B, Suda R, Lai CH (2004). Relationship of *Actinobacillus actinomycetemcomitans *serotype b to aggressive periodontitis: frequency in pure cultured isolates. J Periodontol.

[B47] Leung WK, Ngai VK, Yau JY, Cheung BP, Tsang PW, Corbet EF (2005). Characterization of *Actinobacillus actinomycetemcomitans *isolated from young Chinese aggressive periodontitis patients. J Parodontal Res.

[B48] Nonnenmacher C, Dalpke A, Mutters R, Heeg K (2004). Quantitative detection of periodontopathogens by real-time PCR. J Microbiol Methods.

[B49] Cortelli JR, Cortelli SC, Jordan SH, Zambon JJ (2005). Prevalence of periodontal pathogens in Brazilians with aggressive or chronic periodontitis. Journal Of Clinical Periodontology.

[B50] Program "HYTHER". http://ozone.chem.wayne.edu/.

[B51] BLAST. http://www.ncbi.nlm.nih.gov/BLAST/.

